# Adverse Events Related to Direct-To-Consumer Sequential Aligners—A Study of the MAUDE Database

**DOI:** 10.3390/dj11070174

**Published:** 2023-07-17

**Authors:** Priyanka Belgal, Sahil Mhay, Vrunda Patel, Romesh P. Nalliah

**Affiliations:** School of Dentistry, University of Michigan, Ann Arbor, MI 48109, USA; bpriyanka7@gmail.com (P.B.); sahil_mhay2000@yahoo.com (S.M.); vrunda.patel1692@gmail.com (V.P.)

**Keywords:** adverse events, direct-to-consumer, MAUDE, sequential aligners

## Abstract

Background—Direct-to-consumer (DTC) sequential aligners promote “teeth straightening” at a low cost and with added patient convenience. DTC sequential aligners have risen in popularity among the general public and sparked debate among dental professionals. Dental professionals argue that using these aligners without an in-person diagnosis and treatment planning protocol set by a licensed dentist or orthodontist may lead to adverse effects on teeth and surrounding structures. The objective of this study is to describe adverse clinical events associated with the use of DTC sequential aligners as reported in the Food and Drug Administration’s Manufacturer and User Facility Device Experience (FDA MAUDE) database. Methods—We searched the MAUDE database from 1 January 2010 to 31 December 2020 for the product code of ‘NXC’ (sequential aligners). The year, type of adverse event, reporter occupation, and event description were noted. Results—651 reports associated with sequential aligners were found, of which 104 were related to DTC sequential aligners. Fifty-four adverse events were reported in 2019. From the event description, 41.3% comprised bite problems, 29.8% comprised orofacial pain, and 26.6% of patients had some form of periodontal sequelae. Furthermore, 69.2% of the patients followed up after an adverse event with a dentist not associated with DTC aligners. Conclusions—The use of DTC sequential aligners without dental supervision has led to oral health problems, as documented in the MAUDE database. Commonly reported adverse events include bite problems, pain, sensitivity, and periodontal disease, and some adverse events are irreversible.

## 1. Introduction

The dental profession is a highly specialized discipline within healthcare that manages diseases, injuries, and conditions that affect the orofacial complex. Recent research has shown that 69.3% of medical schools provided fewer than five hours of oral health training to their future medical doctors [1]. Similarly, there is evidence that physicians’ assistants have limited knowledge of oral health and that hands-on training can improve knowledge [2]. A study on emergency room doctors, where 1.5 million dental-related visits occur across the United States (US) each year, showed that 60% reported “no or insufficient” knowledge of managing oral health emergencies [3]. Suffice it to say that oral health knowledge is extremely limited outside of the dental profession. However, among the offerings related to dental services, there is now a new issue—direct to consumer (DTC) sequential aligners. 

Treatment with DTC sequential aligners involve patients receiving orthodontic-related dental services without direct consultation from a dental professional. This non-traditional pathway for dental care requires the patient to visit one of the DTC stores and have dental impressions taken by a staff member—not an orthodontist or general dentist. Another alternative is that the patients can have an at-home impression kit delivered with instructions on making a dental impression. Patients then receive their aligners by mail within four to six weeks after sending their dental impressions to the DTC manufacturers [4]. These aligners are dated and numbered and need to be worn by the patient as per the company’s customized prescription for the patient [4]. There has been a growth in this type of dental care in the last decade for two main reasons—customer convenience and reduced cost of care [4,5]. Initiatives by DTC sequential aligner companies to market their products as cost-effective and convenient have been cited as the primary reason for the paradigm shift in patients seeking better smiles via DTC orthodontic treatment [6,7]. DTC aligners require one or no scheduled visits (none if impressions are taken at home). After having been exposed to such marketing, patients may believe that DTC sequential aligners move teeth faster than traditional braces, fix mild to moderate malocclusions, and require reduced or no visits for follow-up [8]. Moreover, the majority of insurance companies reimburse DTC sequential aligners, which has made this option more appealing [9].

While patients may not be fully aware of the risks posed by DTC aligners, the dental fraternity, especially orthodontists, has argued that straightening teeth is a medical process that should be performed by a trained professional who has undergone specialist training [10]. One of the main concerns with DTC aligners is that, despite being marketed as “dentist-directed” treatments [11], there is no direct dentist–patient communication in any manner (either online or in person) throughout the procedure [12]. Orthodontic treatment without early clinical or radiographic evaluation, diagnosis, or treatment planning risks neglecting underlying conditions which could result in temporary or permanent complications for the patient. DTC aligner manufacturers state that it is the responsibility of the patient to continue routine dental care with a dental professional during the aligner treatment [13]. To obtain DTC sequential aligners, a patient is required to sign an informed consent form stating that he/she has had a dental checkup performed by a dentist. However, because there is no dentist on site, no such dental evaluation is provided.

The development of DTC aligners and the introduction of non-specialists providing orthodontic care has given potential patients more convenient and cost-effective treatment options for straightening their teeth. There have been reports of a high level of satisfaction among patients using DTC sequential aligners; however, only a few scientific studies have been published on this topic [14]. The levels of satisfaction have varied, as patients who used the aligners for cosmetic corrections such as minor crowding or spacing are more satisfied than patients with bite issues [5]. Any errors in the impression will be carried over to the aligner set, and no clinical or radiographic exams are performed prior to commencement of DTC orthodontic care. Currently there are no studies available in the literature regarding adverse events associated with DTC aligners.

The Food and Drug Administration (FDA) oversees medical and dental devices that are available on the market. The FDA requires that manufacturers, device user facilities, and importers submit certain types of reports for adverse events and product problems involving medical devices [15]. As the FDA relies on the data submitted by manufacturers and not on the pre-market testing by dental care professionals, prospective patients cannot assume that the safety and effectiveness of these devices have been proven [16]. There is a paucity of knowledge about adverse events related to DTC sequential aligners. The aim of this study is to describe the characteristics of adverse events associated with DTC aligners administered without dentist supervision that have been reported to the Manufacturer and User Facility Device Experience (MAUDE). 

## 2. Methodology

Medical device reports (MDRs) of suspected device-related deaths, injuries, and malfunctions are sent to the FDA. The FDA employs MDRs to track the functionality of devices, identify any potential device-related safety issues, and contribute to benefit–risk evaluations of these products. The Manufacturer and User Facility Device Experience Database (MAUDE) keeps track of MDRs submitted to the FDA by both mandatory reporters (manufacturers, importers, and device user facilities) and voluntary reporters (healthcare professionals, patients, and consumers) [17]. Each device has a unique product code that was used to search for adverse events related to it. We used the “NXC” code, which has been assigned to sequential aligners. Sequential aligners are either prescribed by orthodontists or dentists or are sold directly to patients. In our research, exclusion criteria consisted of DTC sequential aligner products that included the active involvement of dentists during the treatment of the patients. Inclusion criteria consisted of reports related to DTC sequential aligner products having no oversight by a dentist. The characteristics captured in the MAUDE database include reporter occupation, report number, the date of the adverse event, the type of adverse event, and a description of the adverse event. From the event description, we were able to derive information on the symptoms shown by patients, as well as to determine if they followed up with a dentist after the event and if they needed any additional treatment. Any financial burden faced by patients is also mentioned in the event description. 

The Committee on Human Subjects Research of the University of Michigan Medical School reviewed our protocol and deemed this study as “not regulated” (HUM00189895).

## 3. Results 

**Figure 1:**
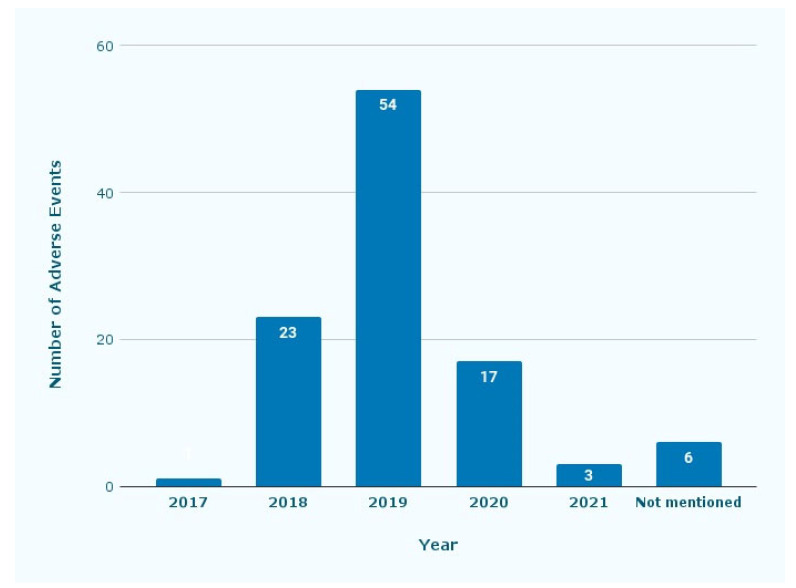
Number of adverse events reported by year. The reports were from 2017 until 2020, with some reports not mentioning which year they were submitted.

Figure 1 shows the year when the adverse events occurred. Fifty-four reports were submitted in 2019, followed by 23 and 17 in 2018 and 2020, respectively. 

**Figure 2:**
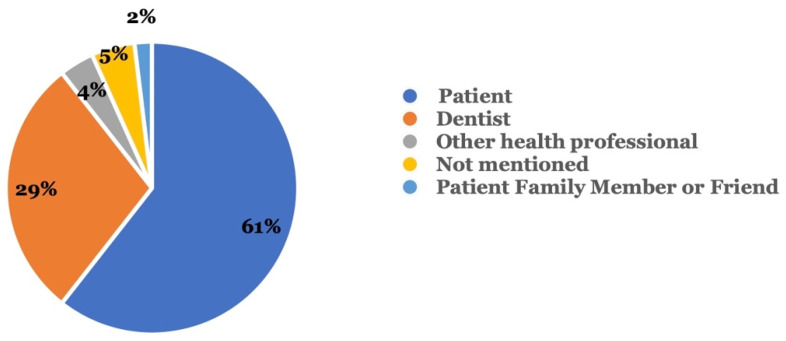
Who reported the adverse event? Reporters included the patient, a dentist, and others.

Figure 2 shows the type of reporter who reported the adverse events. In 60.6% of all cases, the patient reported the adverse event, while dentists were the second most common reporters (28.8%). Other health professionals (3.8%) and patients’ family members or friends (1.9%) constituted a smaller percentage among the reports. 

**Figure 3:**
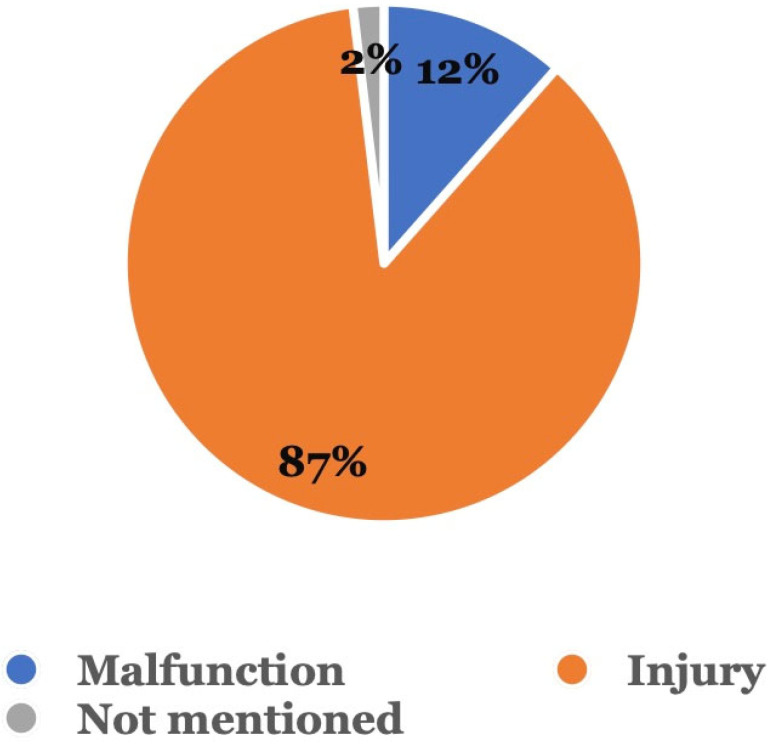
Type of adverse event. Adverse events were categorized as either a malfunction, injury, or other.

Figure 3 shows the type of adverse event that occurred. Among the 104 adverse event reports related to sequential aligners without dentist supervision, 90 (86.5%) were injury reports, followed by 12 (11.5%) involving malfunctions; in 2 (1.9%) cases, the type of event was not mentioned. 

**Figure 4:**
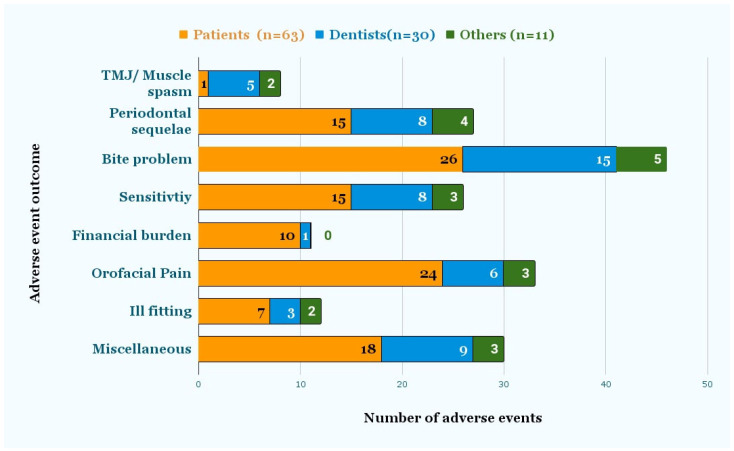
Adverse event outcomes as reported by Dentists and Patients. The signs and symptoms were reported by either the patient or dentist, as recorded in the event description.

Figure 4 shows the signs and symptoms reported by patients, dentists, and others. Symptoms included TMJ/muscle spasm, bite problem, pain, sensitivity, ill-fitting, and periodontal sequelae, such as mobility, recession, and bone loss. Miscellaneous symptoms included caries, fracture, tooth loss, cold sore, dry mouth, irreversible pulpitis, and headaches. According to 63 patient reports, “bite problems” (26) were the most common injury, followed by “orofacial pain” (24), miscellaneous symptoms (18), “periodontal sequelae”, (15) and “sensitivity” (15). Seven out of the 63 reports indicated that the aligners were ill-fitting, and one patient had a muscle spasm/TMJ problem. According to the 30 reports submitted by dentists, “bite problems” (15) were followed by miscellaneous (9), “periodontal sequelae” (8), “sensitivity” (8), and “Orofacial pain” (6). Miscellaneous symptoms included “dental caries”, “irreversible pulpitis”, “headache”, “difficulty chewing”, “cold sore”, and “dry mouth”. Ten out of 63 patients stated that using DTC aligners had led to financial burden, since they subsequently needed additional orthodontic or related dental treatment from a dentist. Finally, 31 out of 104 reports mentioned the need for additional treatment, which included correctional orthodontic, pulp, and periodontal treatment. 

**Figure 5:**
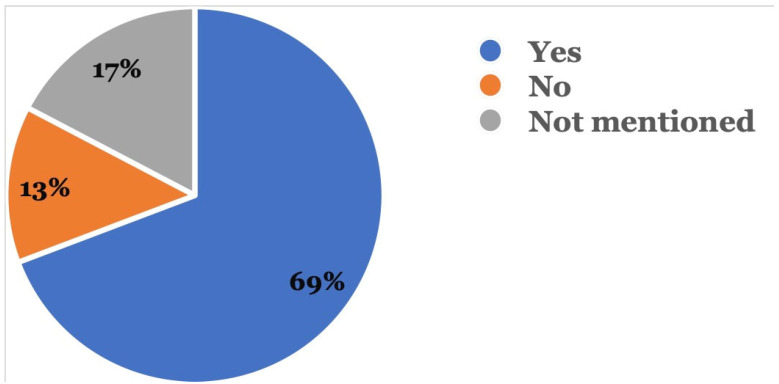
Was there a follow-up with a dental provider? This was recorded in the event description where the reporter mentioned if follow-up was done with a dentist after the adverse event.

Figure 5 shows that in 72 (69.2%) reports, patients followed up with their dental providers after the adverse event occurred, while 18 (17.3%) did not mention if they had followed up with their dentists; 14 (13.5%) patients did not follow up with a dentist after the adverse event occurred.

**Figure 6:**
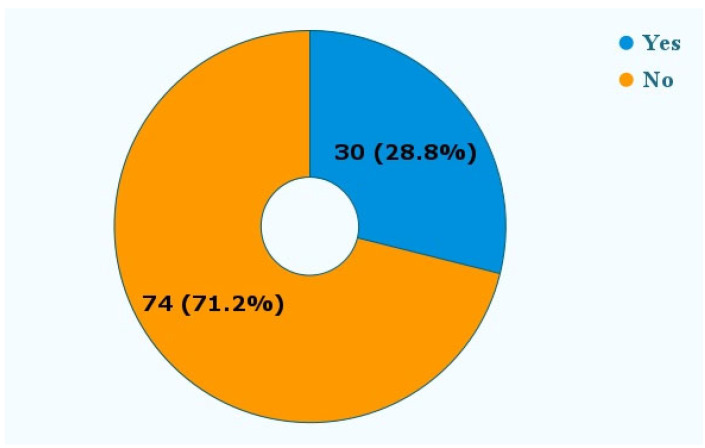
Was additional treatment needed? The reports mentioned the need for additional treatment following the adverse event. These treatments included additional orthodontic therapy, periodontal therapy, and root canal therapy.

Figure 6 shows that in 30 (28.8%) reports, patients needed additional treatment in the form of orthodontic, periodontal, or root canal therapies. 

## 4. Discussion

The concept of using clear aligners in orthodontics has been around since 1946 [18]. Recent technological advancements involving artificial intelligence and rapid prototyping have improved treatment planning and the manufacturing of clear aligners [19,20]. This has also led to an increased number of DTC products and greater accessibility to these products by the general public. The current study found 104 reports of adverse events associated with the use of DTC sequential aligners not supervised by dentists in the MAUDE database during the period of the study (2018–2020)—an average of 34.7 per year. One of the co-authors collaborated in previous research using Invisalign (which involves supervision by clinicians) and found 173 reports over the ten years of that study—an average of 17.3 per year [21]. However, that study of clinician-supervised aligners found more severe adverse events, with 26% deemed to be life-threatening. In the current study of direct-to-consumer aligners, although some cases were severe, no life-threatening conditions were reported.

DTC sequential aligners have been rapidly growing in popularity since 2014, and the first report in our study was submitted in 2017 [22]. Our first paper related to sequential aligners was published in 2017; that report may have had the effect of raising awareness among the dental profession of the MAUDE database [21]. Most of the reports during our study period were reported in 2018–2019, however, reporting decreased during the COVID-19 pandemic.

Out of the 104 reports, 30 were submitted by dentists. Reports related to dental devices submitted by dentists in the MAUDE database were relatively sparse, i.e., only 8% of the total [23]. It is possible that adverse events are underreported because it is not common for dentists to report to or engage with MAUDE. Heballi et al. suggested that the underreporting of adverse events in MAUDE is because of a lack of awareness among dentists and patients about the existence of a public reporting system, as well as the difficulty in submitting a voluntary report [24].

In our study, about 60% of reports were submitted to MAUDE by patients, while 30% were from dentists not involved in the DTC aligner treatment. The dentists involved encountered patients who had sought their services after an adverse event. This could be because many adverse events necessitated these patients to follow up with a dental professional, who then reported these adverse events. In contrast, in the survey done by Wexler, 53.80% of the participants had visited the dentist before starting their aligner treatment, and in 7.90% of cases, the patients were advised against using DTC aligners [8]. Our study adds weight to previous research and underscores the importance of involving dental professionals in the decision-making process for sequential aligner treatment, as trained professionals play a crucial role in identifying potential risks and ensuring patient safety.

According to our findings in Figure 3, the most common adverse event (86.5%) was “injury”。 Although no life-threatening outcomes were reported, injuries to the oral cavity, especially those affecting aesthetics and function, could be debilitating for the patient. In a study done by Wexler et al., where 475 participants were surveyed based on their experience with DTC aligners, 357 had at least one adverse event [8]. 

The most common injury reported by patients was a bite problem, followed by pain, periodontal sequelae, and sensitivity. These findings on adverse events were similar to those reported in a survey of people who had used DTC aligners, though in that study, pain was more common than bite problems.

Despite the reported bite problems caused by DTC aligners, issues related to bite are not common sequelae of traditional orthodontic treatment [25]. Traditional orthodontic treatments have also been associated with outcomes such as periodontal damage, pain, root resorption, tooth devitalization, temporomandibular disorders, speech problems, and enamel damage [26]. However, what is important is that traditional orthodontics includes regular checks by a licensed orthodontist who may be able to identify and resolve these concerns early. 

Wexler et al. reported that 6.6% of their study participants that had used DTC aligners had adverse events which were serious enough to necessitate a dental visit [8]. We found a larger proportion of patients that visited a dentist after the adverse event occurred. In the current study, 69.2% followed up with a dentist following the adverse event while 13.5% did not. In the remaining 17.3% of reports, no follow-up with a dentist was mentioned. Among the 13.4% which did not need a dentist follow-up, reports described miscellaneous occurrences such as ill-fitting aligners, contamination of the aligners, and the use of the wrong set of aligners, which could not have been resolved by a dentist. 

Approximately one-third of the reports in our study, as shown in Figure 6, mentioned that the patients required additional treatment, such as orthodontic or periodontal therapy. While DTC companies, like other orthodontic companies, use a set of novel algorithms to create customized treatment plans, licensed dentists are not involved in the process of reviewing and approving each product before it is sent to the “customer” [20].

As mentioned before, cost and convenience have been the major reasons for preference changes among patients concerning orthodontic treatment [5,6]. While aligners prescribed by dentists and orthodontists may cost up to $6000, DTC companies offer services at one third the price [4,27]. Moreover, traditional orthodontics are partially reimbursed by insurers [28], and DTC sequential aligners are no different [28,29,30]. Although DTC aligners are less expensive, it is important to consider that regular professional reviews are not a part of the treatment plan, which means there is no way to professionally identify an issue at an early stage. It may be problematic that the identification of a treatment issue may be retrospective, i.e., only after a major issue has already occurred. 

For DTC sequential aligners, the evaluation of outcomes of care occurs informally by the patient, which is problematic. A dental professional would combine patient-reported outcomes with a clinical evaluation for a more comprehensive assessment of care. Our research revealed that 11 out of the 104 reports (as seen in Figure 4) noted financial burden due to adverse events caused by DTC sequential aligners. As a result of these adverse events, patients had to undergo supplementary orthodontic or dental procedures, incurring additional expenses. 

The promise of getting the treatment done faster, as advertised by DTC companies, combined with one or no office visits, could also be associated with more people opting for these aligners [31,32]. Advertisements followed by word-of-mouth seems to be the most effective method to recruit patients for DTC aligners; a person is more likely to opt for DTC aligners if they know a friend or relative who has undergone the same treatment [32].

Historically, patients have not had the option of seeking treatment from anyone other than someone licensed to practice dentistry or orthodontics. In a study conducted by Bous et al., 1441 participants were surveyed about their views regarding DTC aligners and traditional orthodontic treatment. About 30.0% of the participants were not aware that orthodontists offer clear aligners [33]. The study also revealed that those who were highly interested in getting their treatment opted for an orthodontist, while those who were moderately interested chose DTC aligners [33]. DTC sequential aligners may be perceived to be more convenient to patients in terms of the overall reduced treatment cost, payment plans, and remote monitoring [31].

However, DTC aligners also introduce some risk of adverse outcomes that are particularly concerning when a case is not actively and consistently being reviewed by a clinician. 

Our study of the MAUDE database has several limitations. First and most importantly, documenting adverse events in MAUDE is not mandatory [32], which may result in underreporting. Secondly, knowledge about the existence of the MAUDE database may be limited, which means that adverse outcomes of DTC may be severely underestimated. Additionally, there is variability among reporters (from patients to dentists to other clinicians), which means there will be variability in reporter knowledge about oral health and dental treatments—this may affect the quality of reports. 

## 5. Conclusions

The use of DTC sequential aligners without supervision by a licensed dental professional can lead to adverse oral health outcomes, some of which are irreversible. Commonly reported adverse events include bite problems, pain, sensitivity, and periodontal disease. New ways to incorporate dentist oversight, perhaps using tele-dentistry, for affordable DTC aligners may reduce barriers to care. 

This research did not receive any specific grant from funding agencies in the public, commercial, or not-for-profit sectors.

## Data Availability

MAUDE data is publically available.
